# A green approach for dyeing cotton fabrics using synthesized reactive disperse dyes and their mixtures under supercritical CO_2_ medium

**DOI:** 10.1038/s41598-024-77606-0

**Published:** 2024-11-06

**Authors:** Hanan Elsisi, Shahinaz Abouelenin, Tarek Abou Elmaaty, Elham Negm

**Affiliations:** 1 Department of Textile Printing, Dyeing & Finishing, Faculty of Applied Arts, Damietta University, Damietta, 34512 Egypt; 2 Chemistry Department, Faculty of Science, Damietta University, New Damietta, 34517 Egypt

**Keywords:** Chemistry, Materials science

## Abstract

Dyeing natural fabrics using supercritical carbon dioxide is challenging, especially without essential color hues. This work demonstrated that two newly developed reactive disperse dyes with distinct colors and shades were generated, one of which featured from the anthraquinone family and the other yellow, containing a pyrazole moiety. These new dyes and their combinations were used to dye cotton fabric using supercritical carbon dioxide and the highest *K/S* values were achieved at 8.73 for the mixture of (blue dye: yellow dye 80:20), however the lowest *K/S* was observed at 7.71 for (blue dye: yellow dye 20:80). The new dyes’ chemical compositions were identified using elemental and spectroscopic analyses. The effectiveness of these dyes and their mixtures for cotton dyeing was discussed. The dyed samples were tested for color fastness, and the results indicated that they had excellent color retention and were highly durable in washing. The increasing patterns in both dyeing rate and build-up curves show good compatibility. Furthermore, desirable shades of green can be achieved by mixing blue and yellow dyes at various ratios in supercritical CO_2_. The compatibility test involves calculating color difference index values for dyed cotton fabrics by utilizing various ratios of a binary mixture of dyes. Furthermore, the dyes under study and dyed samples displayed superior antibacterial properties against gram-positive and gram-negative bacteria compared to certain antibiotics used as a control. These results aligned with the quality and eco-friendly standards required by the industry without the use of water.

**Supplementary Information:**

The online version contains supplementary material available at 10.1038/s41598-024-77606-0.

## Introduction

The traditional water-based dyeing and finishing procedure necessitates a substantial volume of water for the coloring of textile materials and generates heavily polluted water that contains dyestuffs, various chemical additives, as well as auxiliaries that consume high energy^1–3^. The release of this coloring wastewater into the environment results in a dangerous carcinogenic impact on living beings and contributes to a huge pollution problem worldwide^4^. Supercritical fluid dyeing (SFD) technology does not produce industrial wastewater compared to conventional water-bath dyeing. Instead, it offers a dry, environmentally friendly, cost-effective dyeing method^5^. Furthermore, the most commonly used dyeing medium is supercritical carbon dioxide (scCO_2_) due to its environmentally friendly characteristics, including being non-flammable, having low surface viscosity, being non-toxic, having the ability to be recycled, and being cost-effective^6^. Carbon dioxide additionally has a low critical temperature (Tc = 31.1 °C) and a low critical pressure (Pc = 7.38 MPa)^7^. Various synthetic fabrics were effectively dyed using disperse dyes, including polyester, polyamide, and polypropylene in the scCO_2_ dyeing medium, and some studies met the demands of the marketplace^7–13^. Nevertheless, scCO_2_ dyeing of natural fabrics, primarily cotton fabrics, is still challenging since the hydrophobicity characteristics of scCO_2_ make it difficult for cotton fabrics to swell, which are distinguished by the hydrophilicity of their hydroxyl groups. As a result, scCO_2_ cannot weaken the hydrogen bonds between neighboring molecules, disrupting the cotton’s arrangement and hindering the spread of dye throughout the fibers^14^. Furthermore, commercial dyes for cotton are essentially salts that show insolubility in scCO_2_ dyeing. Fortunately, as revealed by our prior research^15–17^ in conjunction with the outcomes of other studies^18–20^, the employing of reactive disperse dyes could fulfill the majority of the requirements of both the scCO_2_ medium and natural fabric throughout scCO_2_^20^. However, producing new colors of cotton fabrics employing scCO_2_ dyeing remains a challenge, as a limited color was reported in previous studies. Consequently, there is an urgent demand to produce dyed cotton with new colors using scCO_2_ dyeing, which can be achieved by mixing different colors of dyes to generate new colors, tunes, and shades of dyed fabric, and this immediately affects the industrialization process of scCO_2_ dyeing. Nevertheless, this particular subject is seldom investigated in the academic literature, as the compatibility of dyes in admixture in scCO_2_ is, therefore, of great practical interest and challenge and rare to be obtained^21^. It is also a foundation for achieving acceptable color-matching productions^22^. On the other hand, the multistep scCO_2_ matching method to dye the fabric greatly extends the dyeing time and increases procedure complexity, reducing the process prospect due to high dyeing expenses. Establishing a more effective matching of colors strategy was reported using one-bath scCO_2_ color matching of polyester fabrics with the mixture of Disperse Red 54, Disperse Blue 79, and Disperse Red 167 dyes^21^. Using waterless beam dyeing in scCO_2_, Wang et al.^23^ reported polyester fabric color matching by mixing Disperse Red 54, Disperse Red 167, and Disperse Blue 79 disperse dyes with high compatibility. The color-matching algorithm of scCO_2_ was enhanced, and an essential database of the adopted disperse dyes in scCO_2_ was constructed to establish the data basis to continue boosting scCO_2_ computer color-matching technologies. Zhao et al.^24^ studied the uptake behaviors and compatibilities of nine developed special disperse dyes that belong to various temperature groups for color matching on polyester fabric using scCO_2_, along with other essential foundations for the application of the scCO_2_ technique. Although the color matching in scCO_2_ using blended dyes was documented only for synthetic fabric, mostly on polyester, no studies have been reported for natural fabrics. Anthraquinones are an important class of organic compounds utilized to produce blue dyes. Furthermore, it is stable in various organic solvents during calibration investigations. It is also a flexible chromophore that can create numerous reactive functional groups^25–28^. Considering all the above, the development of novel blue reactive disperse dye was the main focus of this study to obtain good dyeing performance and color fastness for dyeing cotton fabrics. In the present paper, we developed new reactive disperse dyes based on anthraquinone with reactive functional groups, including vinyl chloride and cyanuric chloride. On the other hand, 5-Aminopyrazoles play a crucial role as a heterocyclic class known for their biological and pharmacological properties. In addition, these compounds exhibit anti-inflammatory, herbicidal, fungicidal, bactericidal, and antipyretic activities^29–33^, and have emerged as promising candidates in the field of dyes due to their versatile chemical properties and vibrant color capabilities. These compounds, characterized by the presence of an amino group and a pyrazole ring structure, offer a unique molecular architecture that allows for tailored modifications to achieve desired dyeing properties. The amino group provides sites for functionalization, enabling the introduction of various substituents to fine-tune the dye’s solubility, stability, and affinity for different substrates. Additionally, the conjugated π-electron system within the pyrazole ring contributes to the dye’s coloration by facilitating electronic transitions, leading to a spectrum of vivid hues. Vinyl sulfone is a reactive group widely utilized in the dyeing of cotton textiles due to its exceptional affinity and permanence. This reactive group, characterized by its vinyl group and sulfone moiety, forms covalent bonds with the hydroxyl groups present on cellulose fibers under mild alkaline conditions^34^. The vinyl sulfone group undergoes nucleophilic addition reactions with the cellulose hydroxyls, resulting in the formation of stable ether linkages. This covalent attachment ensures excellent wash fastness and resistance to chemical degradation, making vinyl sulfone-based dyes ideal for applications requiring durability and color fastness, such as apparel and home textiles. The main aim of this research is to analyze the effectiveness of two newly developed special reactive disperse dyes in achieving color consistency on cotton materials in scCO_2_. It also aims to dye cotton fabrics with various shades using supercritical coloration technology. Various proportions of reactive disperse dye mixtures were used in a single-bath color-combination process for cotton. The characteristics and compatibility of blue and yellow dye were investigated in scCO_2_. In addition, practical new color mixtures were carried out on cotton fabric in scCO_2_, focusing on their leveling properties and color fastness. In summary, this research significantly advances the dyeing process of cotton fabrics using scCO_2_.

## Experimental

### Materials

Shikisen-sha Company in Osaka, Japan, provided knitted textiles made from cotton with specific details including 750 m1 twist, 28 Ne count, 9.21% U%, 11.70 CVM, 0 thin − 50%, 33.6 thick + 50%, 29.8 Neps + 200%, and 63.4 total imperfection percentage. The fabric used is composed of single jersey weft knit cotton and is produced using a single row of needles. One side of the fabric features flat loops, while the other side has a reverse loop structure.

All reactant chemicals were obtained from Sigma Aldrich without any additional purification steps. The solvent used for the supercritical fluid Dyeing was CO_2_ (99.6%, supplied by NETCO Industrial Company, Cairo, Egypt). The solvent used to synthesize dyes was from the Faculty of Applied Arts, Damietta University, Egypt.

## Apparatus

The original values for melting points in Celsius were measured using open capillary tubes and a Griffin melting point apparatus. The^1^H and^13^C-NMR spectra were obtained using a Bruker Avance III 400 MHz NMR spectrometer and a JEOL ECA-II 500 MHz NMR spectrometer, with CDCl_3_ and DMSO-d_6_ as solvents. Infrared spectra were produced for KBr pellets using a JASCO 410 spectrometer, with specific absorptions noted within the 4000 –400 cm^− 1^ range. Chemical shifts were referenced in (ppm) relative to tetramethyl silane. Mass spectra were collected using MS equipment with 70 eV (Kratos) and a Varian MAT 311 A Spectrometer. Analytical thin-layer chromatography utilized 0.2 mm pre-coated silica gel G60 F plates from Merck, visualized under UV light (254 and 366 nm) or iodine vapor.

## Dye synthesis and analysis of its chemical composition

### Synthesis of 1, 5-diamino-4-((4-((4, 6-dichloro-1, 3, 5-triazin-2-yl)-amino)-phenyl)-amino)-8-hydroxyanthracene-9, 10-dione 3: (Blue Dye)

**Scheme 1:**
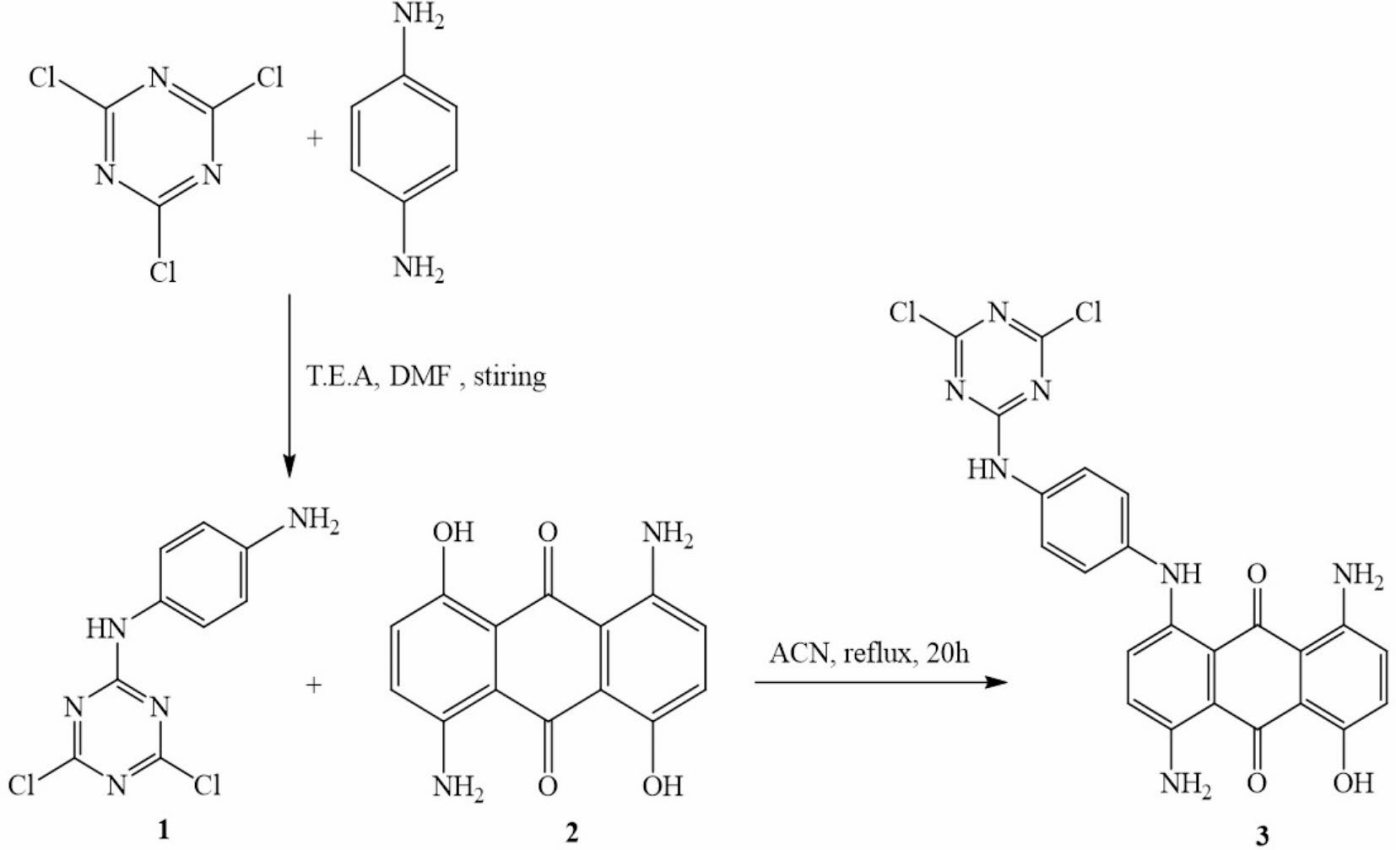
Synthetic routine of blue dye.

In dimethylformamide (50 ml), a solution containing p-phenylenediamine (10 mmol) was mixed with Et3N (8 mmol) and then with 2, 4, 6-trichloro-1,3,5-triazine (12 mmol). The resulting mixture was stirred at room temperature for 12 h. When the reaction was complete, the solution was poured into crushed ice to obtain a precipitate, filtered, and dried to obtain the final product 1. Next, a mixture of 1, 5-diamino-4,8-dihydroxy anthracene-9,10-dione (2) (5 mmol) and compound 1 (5 mmol) was dissolved in acetonitrile (40 ml) and refluxed. After 20 h, the acetonitrile solvent was evaporated under reduced pressure as illustrated in Scheme 1. The crude reaction mixture was purified by adsorbing it onto silica gel (230–400 mesh) and subsequently subjected to column chromatography. Elution was performed using a gradient of ethyl acetate: n-hexane, ranging from 1:4 to pure. This separation process resulted in the isolation of compound **3** as a pure blue solid.65% yield, its maximum absorption wavelength (λ_max_) in DMF was 613.5 nm. IR (ν/cm^− 1^): 1115.84 (C-Cl), 2050.84 (N = C = N), 1605 (C = C_aromatic_), 1634 (C = O), 3329 (NH), 34,152 − 3405 (NH_2_), 3540.84 (OH);^1^H NMR (500 MHz, CDCl_3_): δ 4.32 (S, 2 H, NH_2_ ), 6.75 (d, 2 H, H_aromatic_), 7.05 (S, 2 H, NH_2_), 7.16 (d, 1 H, H_aromatic_), 7.34 (d, 1 H, H_aromatic_), 7.5 (d, 4 H, H_aromatic_), 8.2 (s, 1 H, NH),12.3 (s, 2 H, NH), 13.82 (s, 1 H, OH)^13^. CNMR (CDCl_3,_ 500 MHz): 114.59, 118.4, 120.4, 122.774, 126.995, 128.221, 128.412, 129.995, 133.801, 137.7, 144.2, 145.819, 152.334, 155.72, 156.435, 159.5, 169.5, 183.3(C = O). MS (ESI) m/z Calc. for C_23_H_15_Cl_2_N_7_O_3_ (508) found 510 [M]^+2^.

## Synthesis of compound 5

A combination of 3-(3-chlorophenyl)-2-azo-3-oxopropanenitrile **4** (10 mmol) and hydrazine hydrate (15 mmol) were added to a round bottom flask and heated at 100 ºC for 5 h. The advancement of the reaction was monitored through TLC using EtOAc/PE (ratio 1:3) as the eluent system. After cooling, the reaction mixture was poured onto crushed ice. After the formation of the precipitate, it was separated by filtration and subsequently washed with water to yield product **5** in the form of a yellow powder. 87% yield, m.p (200–202 °C); IR (ν/cm^− 1^): 1481–1490 (N = N), 1601 (C = C_aromatic_), 3175 (NH), 3288–3450 (NH_2_);^1^H NMR (CDCl_3_, 500 MHz): δ 6.5 (s, 2 H, NH_2exchange with D2O_), 7.28–7.33(d, 2 H, H_aromatic_), 7.416 (d, 2 H, H_aromatic_), 7.505 (S, 1 H, H_aromatic_), 7.66 (d, 2 H, H_aromatic_), 7.708 (s, 1 H, H_aromatic_), 10.9 (s, 1 H, NH_exchange with D2O_ );^13^C NMR (CDCl_3,_ 125.6 MHz): δ 86.6, 125.855, 128.164, 129.28, 129.3, 129.54, 130.5, 130.66, 130.75, 134.29, 135.36, 138.322, 141.89, 146.906, 151.34. MS (ESI) m/z Calc. for C_15_H_11_C_l2_N_5_ (332). Found 331 M^+^.

### Synthesis of N-(3-(3-chlorophenyl)-4-((4-chlorophenyl) diazenyl)-1 H-pyrazol-5-yl) ethane sulfonamide (6)

**Scheme 2:**
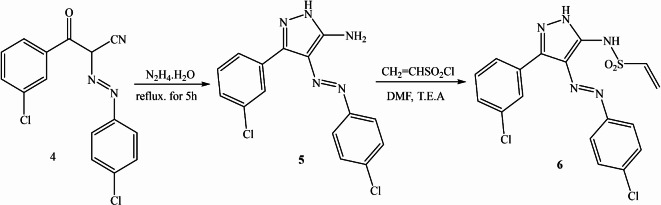
Synthetic routine of Yellow Dye.

In an appropriate dried solvent N, N-dimethylformamide (DMF 30 ml), compound **5** (0.02 mmol), vinyl sulfonyl chloride (0.02 mmole), and triethylamine (0.02 mmol) were vigorously mixed at 0 °C. The mixture was stirred at room temperature for 4 h. The yellow powder final product **6** was obtained by forming a precipitate by pouring the mixture onto crushed ice, filtering, water-washing, and drying as shown in Scheme 2. Then, crystallization with ethanol. 84% yield, m. p. (234–236 °C); Thin layer chromatography was used for characterization. Rf 0.59 eluent system (ethyl acetate: petroleum ether 1:3 v/v on silica gel). IR (ν/cm^− 1^): 1016 (C-Cl), 1086 (SO_2_), 1191 (C-S), 1369–1480 (N = N), 1609 (C = C_aromatic_), 1668.12 (C = C Vinyl), 3289 (NH), 3315 (NH);^1^H NMR (500 MHz, CDCl_3_): δ 5.599 (d.d, 1 H), 6.4 (d.d, 1 H), 6.8(d.d, 1 H), 7.2(S, 1 H, H_aromatic_), 7.2–7.41(m, 4 H, H_aromatic_), 7.506 (S, 1 H, H_aromatic_), 7.61–7.66 (d, 2 H, H_aromatic_), 8.3 (S, 1 H, NH _exchange with D2O_), 11.5 (S, 1 H, NH _exchange with D2O_)^13^, C NMR (CDCl_3,_ 125.6 MHz): δ 88.5, 123.108, 124.98, 125.855, 128.164, 129.051, 129.28, 130.5, 130.66, 130.75, 134.29, 135.36, 138.322, 141.89, 146.906, 151.34; MS (ESI) m/z Calc. for C_17_H_13_C_l2_N_5_O_2_S (422), found 421.02 (100.0%), 423.01 (63.9%), 422.02 (18.4%), 424.02 (11.8%), 425.01 (10.2%), 423.01 (4.5%), 425.01 (2.9%).

## Dyeing process and the design of experiments under ScCO_2_ medium

Place the purified dyes into the bottom of the dyeing vessel and blend them gently according to the studied mixture and concentration. Then, warp (3 × 10 cm) of the cotton fabric for dyeing around a porous beam and suspend it inside the dyeing vessel. The stainless steel vessel has an internal capacity of 50 ml. The dyeing process method is established according to our previous work^16^.

Compatibility is a crucial factor in assessing the dyeing characteristics of dyes, as it demonstrates how well different dyes are dyed in harmony. Obtaining compound shades on textiles relies heavily on the compatibility between two dyes in a binary mixture, making it essential to avoid using incompatible dyes for this purpose. It can be challenging to control the development of compound shades accurately to achieve a specific color tone due to the varying color build-up rates of different dyes in a mixture. Dyes that have similar or identical exhaustion properties are considered to be compatible with each other. Various methods exist for evaluating compatibility between two dyes in a binary mixture.

However, controlling the color shade can be challenging when dealing with dyes of different dyeing rates, as factors like (1 to 4) % of dye concentrations; pressures of (15, 20, and 25) MPa; dyeing temperatures of (100, 110, and 120) °C; and static periods of (60, 90, and 120) minutes can significantly impact the outcome. Conversely, having dyes with good compatibility ensures that the dissolution, diffusion, and adsorption properties of dyes in scCO_2_ remain consistent, making it easier to achieve the desired color shade. Single-factor experiments were carried out to optimize the scCO_2_ of cotton fabrics using a mixture (50–50%) of the dyes under study.

Different mixtures ranging from (80:20, 75:25, 20:80, and 25:75) % of blue dye and yellow dye were investigated at different dye concentrations and optimum conditions of different factors.

## Assessment of the quality characteristics and compatibility of mixed-dyed cotton fabrics

After scCO_2_ with various dyeing conditions, the color strength value (*K/S*) in the visible region of the spectrum (360–740 nm) was evaluated using the Kubelka–Munk Eq. (1):

1 $$\:K/S\:=\:{\left(1\hspace{0.17em}-\hspace{0.17em}R\right)}^{2}/\left(2R\right)$$

Where K is the absorption coefficient, R is the reflectance of the dyed sample, and S is the scattering coefficient.

To guarantee the precision of the experiment, the colored fabric was folded into four layers. Subsequently, three random test points were measured, and the average value was calculated.

Five measurements of L^*^, representing lightness/darkness; a^*^, representing red/green chromaticity coordinates; b^*^, representing yellow/blue chroma, and hue angle (h^*^) of the dyed cotton fabrics were assessed using a Konica Minolta spectrophotometer (Japan; model CM-3600 A). The total color difference (∆ E) was determined based on the Eq. (2).

2 $$\:\varDelta\:E=\sqrt{{\left[\right(\varDelta\:{L}^{*})}^{2}+{(\varDelta\:{a}^{*})}^{2}+{(\varDelta\:{b}^{*})}^{2}]}$$

To evaluate dye attachment and eliminate excess dye molecules that were not fully absorbed by the substrate during the dyeing process, a portion of dyed cotton fabric was subjected to extraction in a Soxhlet apparatus using a solution containing 50% acetone in water for 30 min. The quantity of dye absorbed by the cotton after the dyeing and extraction processes was used to determine dye fixation according to Eq. (3).

3 $$\:\text{F}=\frac{\left(\frac{\text{K}}{\text{S}}\right)\:\text{e}\text{x}\text{t}\text{r}.}{(\text{K}/\text{S})\:\text{d}\text{y}\text{e}\text{d}}\times\:100\text{\%}$$

The color difference index (CDI) values reflect the impact of color change resulting from variations in dyeing process variables on the significant color difference parameters between the blank sample and the dyed sample when dyed under various conditions using different proportions of a binary mixture of dyes. These values signify the overall distribution of diverse color output and color variances in terms of hue, chroma, and metamerism values. The calculation of the color difference index (CDI) was based on a previously established empirical relationship.

4 $$\:\text{C}\text{o}\text{l}\text{o}\text{r}\:\text{D}\text{i}\text{f}\text{f}\text{e}\text{r}\text{e}\text{n}\text{c}\text{e}\:\text{I}\text{n}\text{d}\text{e}\text{x}\:\left(\text{C}\text{D}\text{I}\right)=\:\frac{\varDelta\:\text{E}\times\:\varDelta\:\text{H}}{\varDelta\:\text{C}\times\:\text{M}\text{I}}$$

The magnitudes of the related ∆E, ∆C, ∆H, and MI values, irrespective of their sign or direction, can be used to calculate the CDI (Color difference index) when different ratios of binary pairs of dyes are applied to the same fabric. A compatibility rating ranging from 0 to 5 indicates the level of compatibility, with a rating of 5 indicating maximum or excellent compatibility, a rating of 1 representing minimal or poor compatibility, and a rating of 0 considered entirely incompatible. The compatibility rating increases as the CDI values for binary dye pairings approach each other.

The CDI index is currently utilized in the relative compatibility rating (RCR) method to assess the compatibility of two dyes in a binary mixture. The variance between the maximum and minimum CDI values serves as a basis for evaluating the color build-up rate in the relative compatibility method. This is done by dyeing with varying proportions of the two dyes in the mixture to determine a numerical rating of compatibility ranging from 0 to 5 in the RCR method^35^.

At optimal conditions of dyed samples, The colorfastness was indicated through AATCC standard methods such as (AATCC-61–2 A-1996) for washing, (AATCC 8–2001) for rubbing^36^, (AATCC-16 A-1972) for light fastness^37^, and (AATCC 15-1997) for perspiration fastness^36^. AATCC Test Method 61 (2 A)-1996 was used to evaluate the stability, rechargeability, and durability of the blended dyes on the cotton surface against repeated washing cycles (up to 5 washing cycles)^17^.

### Antibacterial activity determination of both synthesized dyes and dyed samples

The antibacterial activity of the synthesized dyes was evaluated using the AATCC (147–2004) test; it was expressed as the growth inhibition zone (mm). However, the antibacterial testing of dyed cotton samples was done using the AATCC test method 100:2004 for the quantitative assessment of the antibacterial effectiveness against Gram-positive and Gram-negative bacteria^38^.

### Statistical analysis

The average of three values was used for each measurement. The standard error of the mean was determined using a specific equation and was found to be approximately ± 0.1.

$$\:\text{S}\text{E}=\frac{S}{\surd\:n}$$

Where S represents the sample standard deviation, while n represents the number of observations in the sample.

## Results and discussion

### Chemistry of dyes

Scheme 1 outlines the strategy used to synthesize the target compounds **3**. According to reports, it was produced using various reaction conditions. An equimolar mixture of substituted chemicals could be prepared by refluxing them N1-(4, 6-dichloro-1, 3, 5-triazin-2-yl) benzene-1, 4-diamine 1 with a derivative of anthraquinone **2** using acetonitrile as a solvent, as reported. On the other hand, Scheme 2 compound **6** was reported to be prepared via two steps reaction starting with 3-(3-chlorophenyl)-2-((4-chlorophenyl)diazenyl)-3-oxopropanenitrile 4 preparation, a condensation reaction with hydrazine hydrate in ethanol producing 3-(3-chlorophenyl)-4-((4-chlorophenyl) diazenyl)-1 H-pyrazol-5-amine 5. After that, 3-(3-chlorophenyl)-4-((4-chlorophenyl)diazenyl)-1 H-pyrazol-5-amine 5 was subjected to react with vinyl sulfonyl chloride in dimethylformamide as a solvent, and the presence of triethylamine as a basic catalyst to afford the target compound 6 in excellent yields 84%. The structure elucidation of the newly synthesized compounds was achieved through a combination of mass spectrometry and extensive nuclear magnetic resonance (NMR) studies, including both proton and carbon-13 NMR spectroscopy.

### Dyeing behaviors of the two reactive disperse dyes and their mixtures on cotton substrates in scCO_2_

**Fig. 1:**
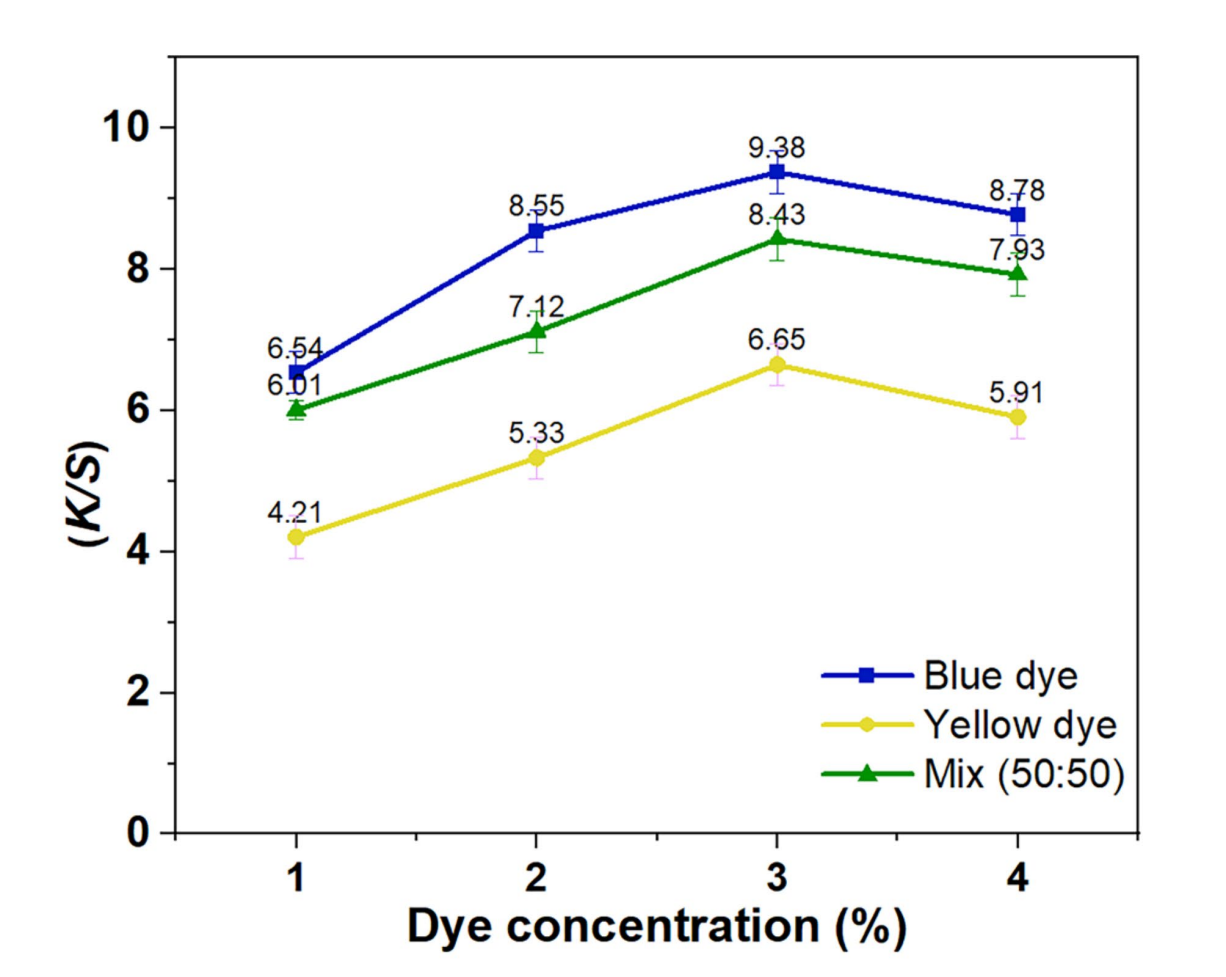
Build-up curves of the two reactive disperse dyes and their mixture (50:50).

The accumulation of both reactive disperse dyes on cotton fabrics can provide a detailed analysis of their coloration, the way they bind to the fiber, and the shade of the dye. The accumulation curves for reactive disperse were examined using scCO_2_ with dye concentrations varying from 1 to 4% (o.w.f.). The results from Fig. 1. show that the *K/S* values of the dyed cotton fabrics increase as the dye concentrations increase. It is also evident that there is a consistent linear increase in *K/S* values when dye concentrations range from 1 to 3% (o.w.f.). Still, no significant improvement is seen when the dye concentration exceeds 3% (o.w.f.). The reason for this is primarily due to the lower level of dye molecule bonding at lower concentrations of dye in scCO_2_. As a result, it is simpler for the dyes to penetrate the cotton fabrics with the movement of the CO_2_ fluid. When the dye concentration is higher, the size of dye particles increases as a result of dye association, making it more challenging for the dye to diffuse from scCO_2_ to cotton fabrics.

**Fig. 2:**
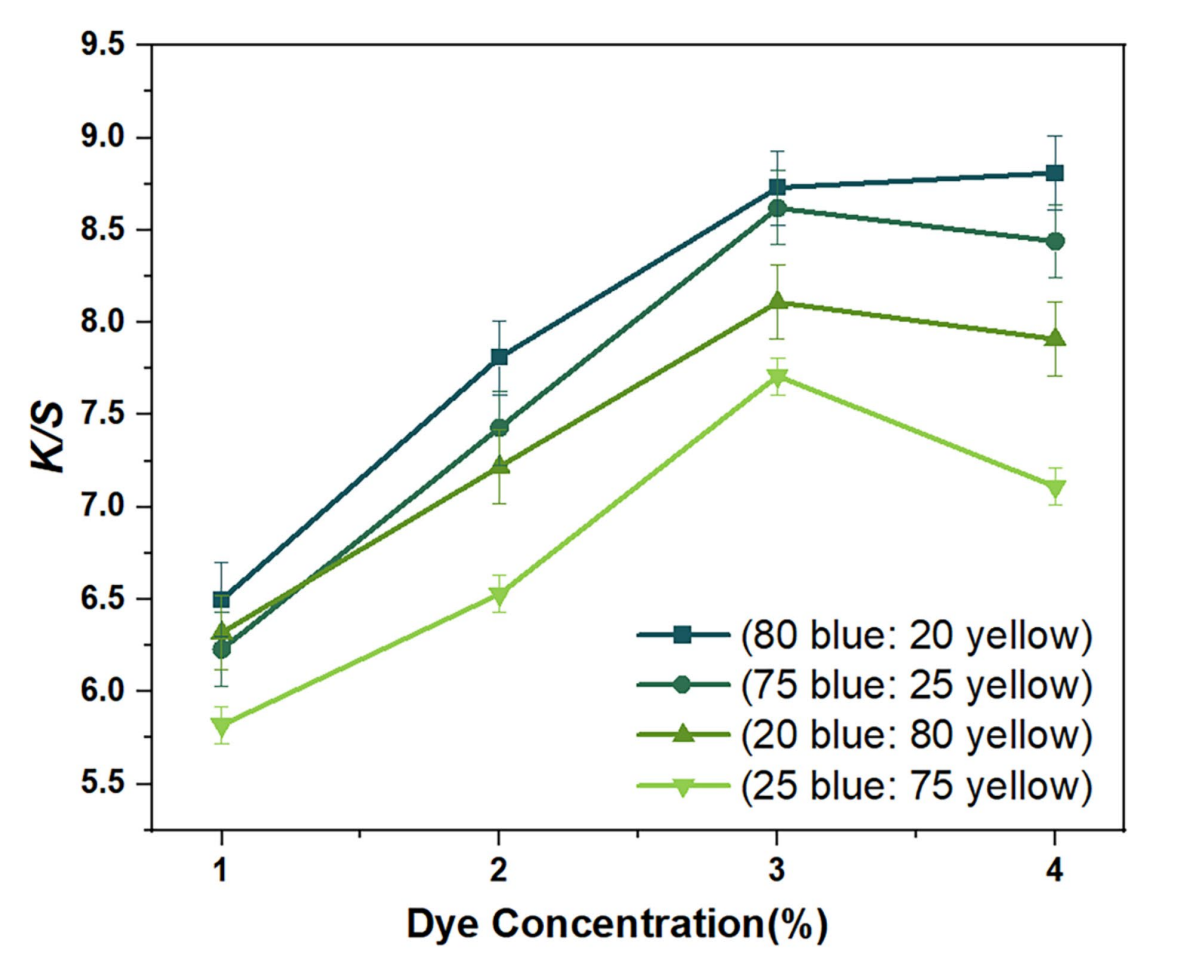
Build-up curves of different mixture ratios of the two reactive disperse dyes.

Figure 2. illustrates the color combination characteristics of blue reactive disperse dye and yellow reactive disperse dye at various dye mixing ratios and concentrations. The results show that all combinations of dyes exhibit good build-up properties in terms of color strength, as indicated by the increase in *K/S* values with higher dye concentrations. As demonstrated in Fig. 2, when blue reactive disperse dye and yellow reactive disperse dye are combined at 80:20, the *K/S* values of the cotton sample gradually improve with the dye concentration increasing from 1% (o.w.f.) to 4% (o.w.f.). However, the application of different proportions (75:25, 50:50, 20:80, and 25:75 blue dye and yellow dye) of binary pairs of the selective dyes on the cotton fabric leads to a significant enhancement in the *K/S* values of the cotton when the dye concentrations increase from 1% (o.w.f.) to 3% (o.w.f.). There is no significant increase in the *K/S* values when the dye concentration is raised to 4% (o.w.f.) because the fibers are already saturated with dye adsorption.

**Fig. 3:**
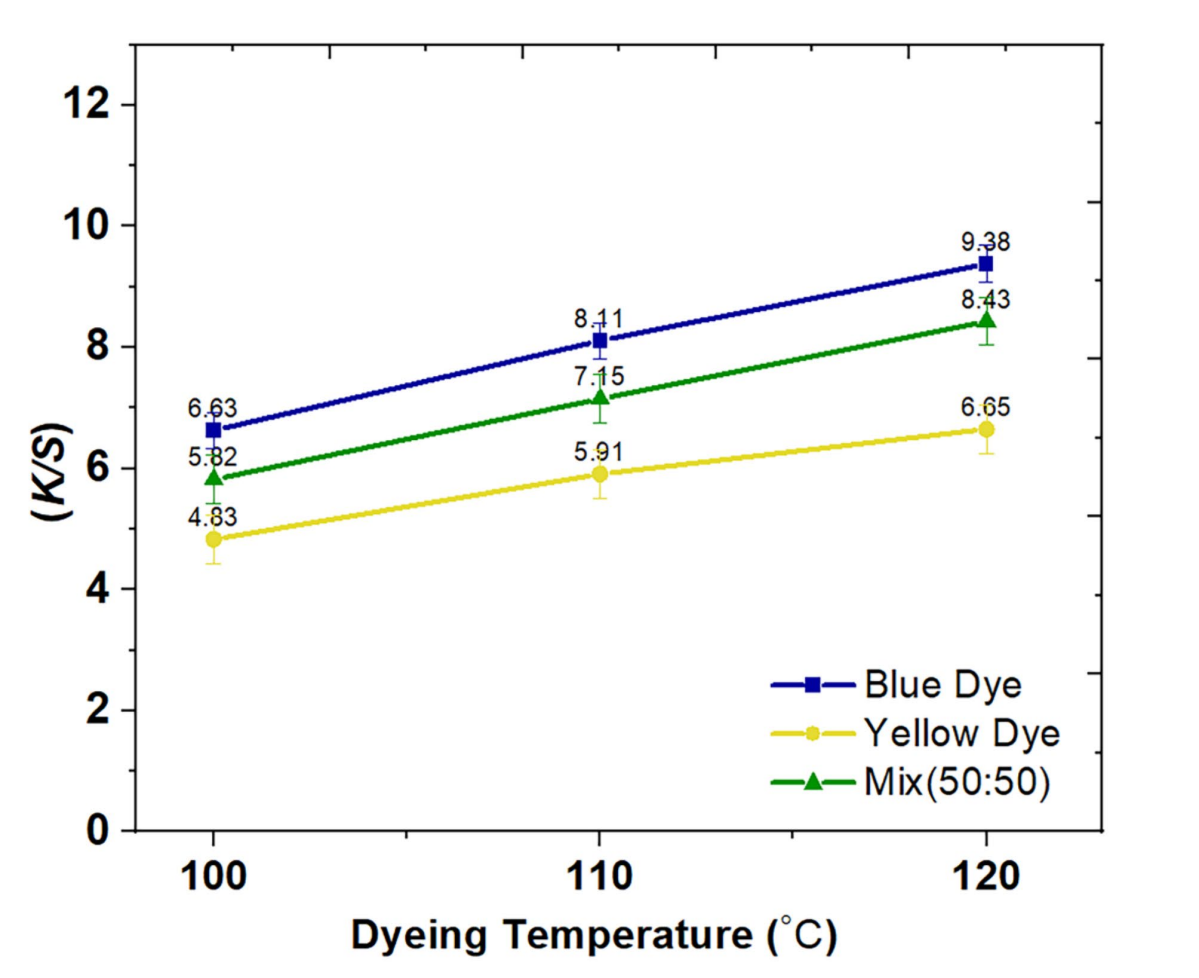
Relationship curves between dyeing temperature and *K/S* for the two dyes under study and their mixture (50:50).

According to Fig. 3, the impact of temperature on the *K/S* of cotton fabric dyed with a 3% dye concentration, 25 MPa pressure, 90 min dyeing time, and system temperatures ranging from 100 °C to 120 °C is demonstrated. The findings have shown that the *K/S* value increases with increasing temperature because higher temperature improves the ability of the dye molecules to penetrate the fabric, creating more extensive pores and increasing diffusion through increased swelling and reactivity of the dye. The dye’s reactivity significantly increased when the temperature rose from 100 °C to 120 °C, as there was a noticeable boost in fixation, indicating the formation of more covalent bonds with the temperature rise.

**Fig. 4:**
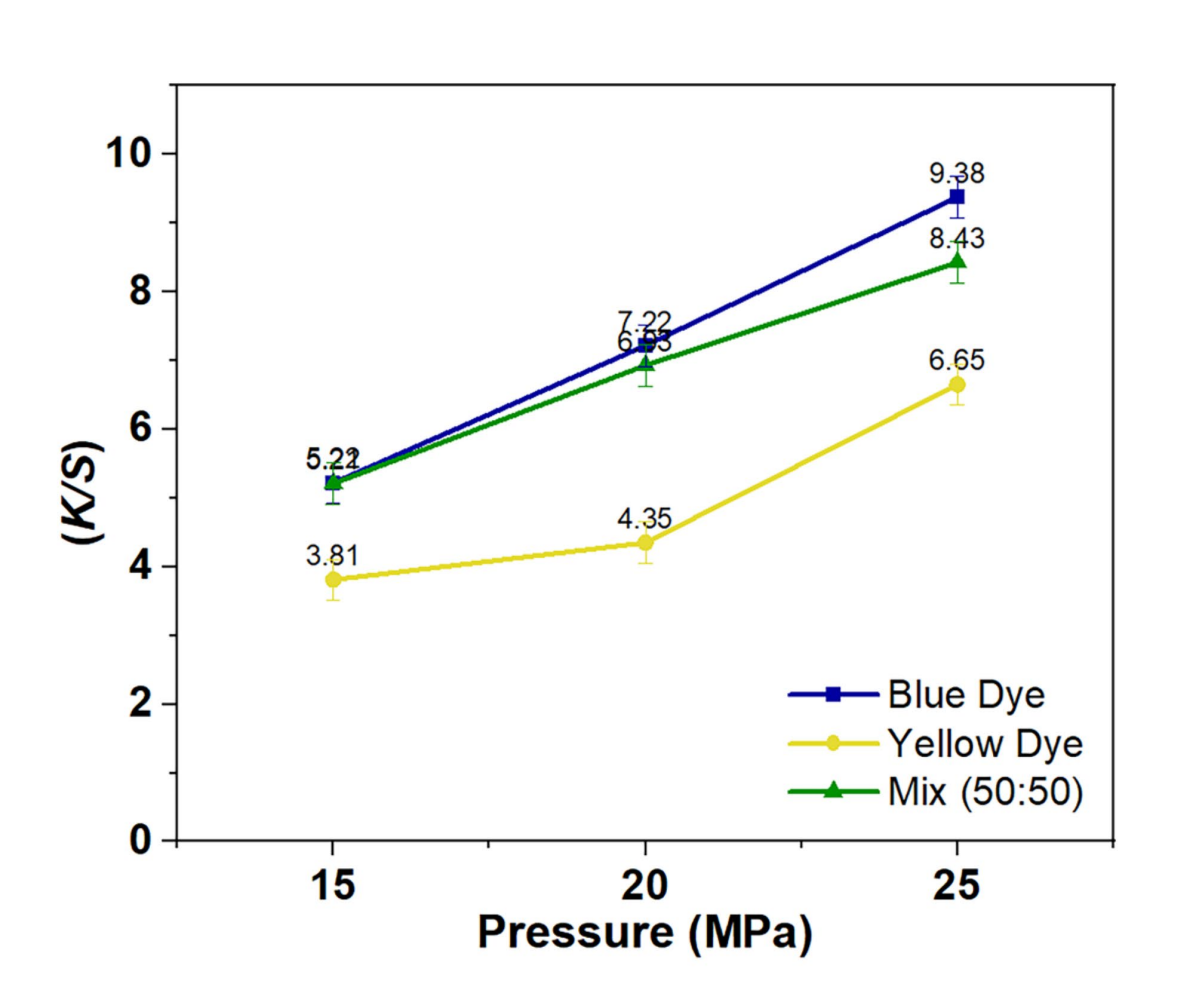
Relationship curves between pressure and *K/S* for the two dyes under study and their mixture (50:50).

To further study the impact of system pressure on the uptake behaviors and color mixture of both reactive disperse dyes. System pressure levels ranging from 15 MPa, 20 MPa, and 25 MPa were studied. The findings of the study are illustrated in Fig. 4, which highlights the significant and diverse impacts of pressures on the absorption behaviors and compatibilities of the two dyes under study. These effects are seen through different and total proportional increases in the curves. Specifically, as the pressure used for dyeing increased between 15 and 25 MPa, there were consistent and corresponding increases in absorption behaviors seen for yellow and blue dyes, as well as their combinations, on the material. The dissolving behavior of dye molecules in the medium is directly influenced by pressure, with all other factors remaining constant. Theoretically, when the pressure during the dyeing process rises, a greater amount of dye molecules dissolve and are found in higher concentrations in the supercritical bulk fluid. This increase enables improved transfer, thermal diffusion, and adsorption of dye molecules on cotton fabrics, resulting in enhanced uptake behaviors of reactive disperse dyes^20,39^.

**Fig. 5:**
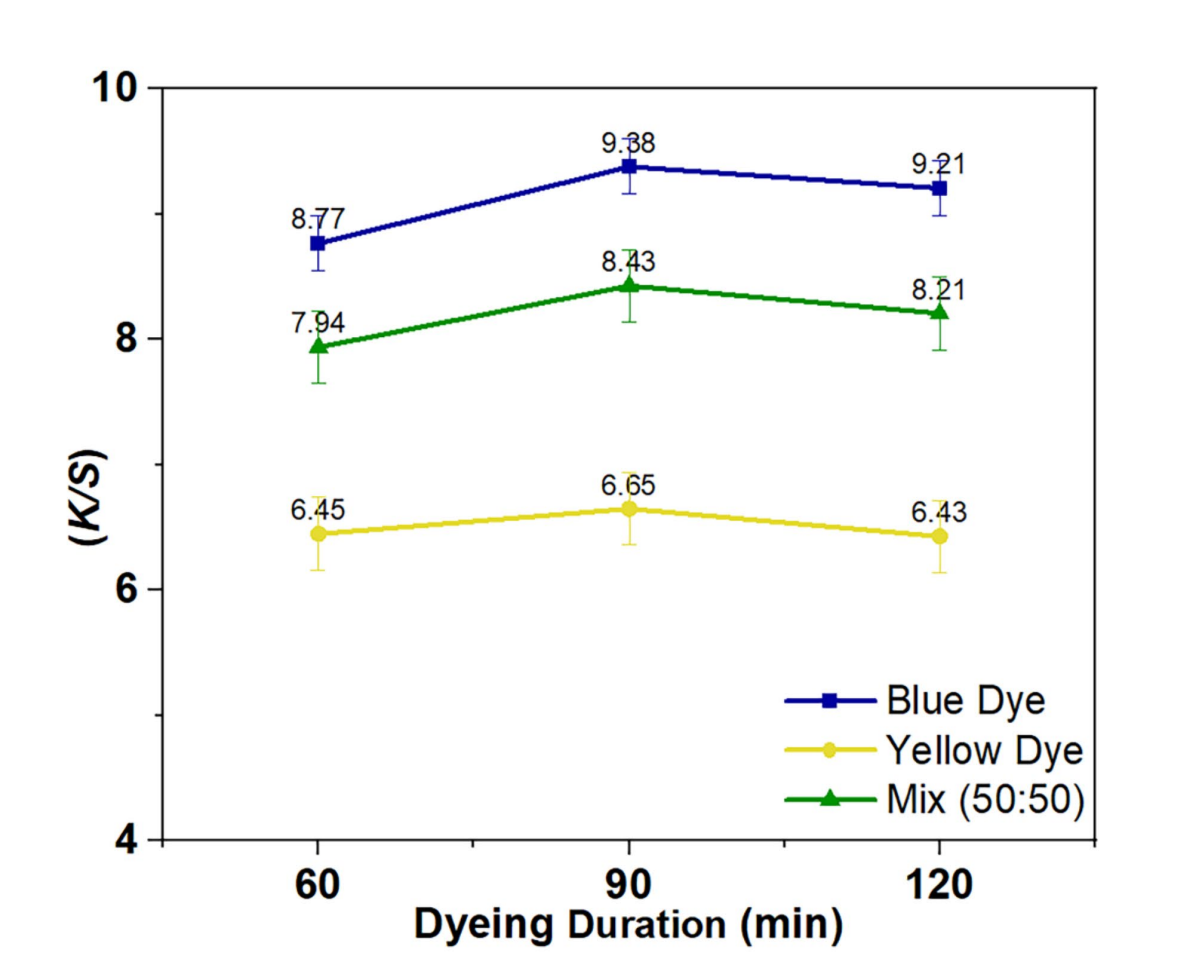
Relationship curves between dyeing time and *K/S* for the two dyes under study and their mixture (50:50).

In the scCO_2_ process of dyeing and color combinations, the duration of dyeing is crucial. Therefore, the study focused on analyzing the effects of dyeing duration on uptake behaviors and the compatibility of reactive disperse dyes under consistent temperature and pressure settings. According to the results shown in Fig. 5, the *K/S* values of cotton substrate enhanced as the dyeing time increased from 60 to 90 min. This significant improvement in value prompted the selection of 90 min as the optimal time for further investigation of other variables. The decision to focus on the 90-hour mark was based on the observation that more reactive dye groups were bound to the fabric molecules at this time, minimizing further absorption between 120 min.

### Compatibility evaluation of dyed cotton samples

The current study also utilized the color difference index (CDI) to analyze how the total differences in color strength are affected by changes in color build-up rate, hue, chroma, metamerism, and proportion of dyes or variations in dyeing process variables. The CDI provides a single index value that reflects the combined impact of various color difference parameters when samples are dyed with different shades under a scCO_2_ medium. Higher CDI values suggest a greater need for precise control of color variation in a specific dyeing condition or when adjusting the individual proportion of dyes in a mixed shade to create complex colors.

Table 1 displays the data for total color differences (∆E), changes in hue (∆H), changes in chroma (∆C), metamerism index (MI), and brightness index (BI) of dyed cotton fabrics concerning blank cotton. The dyeing process involved using a single reactive disperse dye and a combination of both dyes under study, both at similar shade depths and under identical dyeing conditions. The ΔE* value is from 18.53 to 21.99, indicating a significant difference in colors. The ΔE value represents the overall color difference of the colored sample, with higher values indicating a larger deviation from the color of the uncolored fabric and lower values indicating a smaller difference. The brightness index (BI) is a significant color characteristic for dyed fabrics, which is largely influenced by surface shine and specular reflectance. The BI values for these binary dyes are ranked in the order of M1 > M2 > M5 > M3 > M4.

As shown in Table 2, the compatibility grade, as per RCR values, displayed good results. This result could be attributed to the similarity in CDI values for the binary pairs of dye ratios under similar dyeing conditions, (80:20, 75:25, 50:50, 25:75, 20:80), resulting in a higher level of color uniformity and improved dispersion across the cotton fabric. Additionally, the lower the values of (CDI max and CDI min) when dyeing with varying proportions of two dyes in a binary mixture, the stronger the compatibility between them.

**Table 1:** Color parameters of cotton fabrics dyed with a binary mixture of developed reactive disperse dyes.

Dyes- combination	K/S	∆E	∆C	∆H	MI	BI	CDI
M1 (Blue dye: Yellow dye 80:20)	8.73	21.99	16.53	18.32	4.89	9.93	4.80
M2 (Blue dye: Yellow dye 75:25)	8.62	21.03	14.49	16.74	5.07	9.43	4.79
M3 (Blue dye: Yellow dye 50:50)	8.43	21.08	13.27	11.91	4.02	6.92	4.70
M4 (Blue dye: Yellow dye 25:75)	8.11	18.53	14.10	18.47	5.23	5.43	4.64
M5 (Blue dye: Yellow dye 20:80)	7.71	21.54	16.55	17.55	4.9	7.43	4.66

**Table 2:** Color difference index and compatibility grade of mixed dyed cotton samples.

Binary pair of dye combination	CDI values					CDI max – CDI min difference	RCR	Compatibility grade as per RCR values
	80:20	75:25	50:50	25:75	20:80			
Blue dye: Yellow dye	4.80	4.79	4.70	4.64	4.66	4.80–4.64 = 0.16	4	Good

### Color performance of dyed cotton samples

**Fig. 6:**
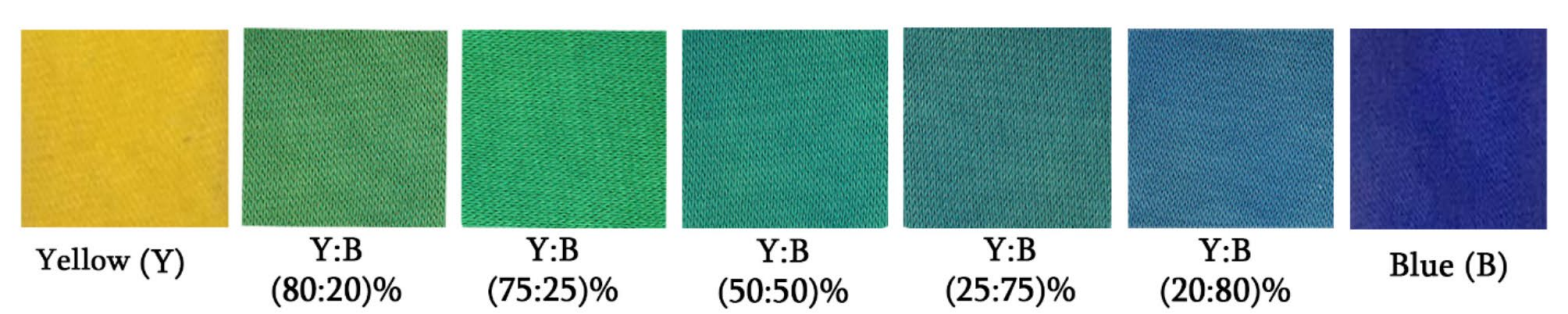
Digital picture of dyed mixed samples.

As illustrated in Table 3, for the blue sample, the color lightness value (L^*^) recorded was 24.22, which shifted towards darkness. However, the yellow sample is shifted towards whiteness (89.5). The L^*^ values of the mixed dyed cotton samples ranged from 45.11 to 60.29, with L^*^ representing the brightness or transparency level in color. Pure black is represented by L^*^ = 0, and pure white by L* = 100. Sample (20B-80Y) is lighter than the other mixed samples. The color tones of the mixed dyed samples are inclined in a greenish direction on the red-green axis due to the negative values of a^*^. The negative values of b^*^ shift the color tones of all mixed samples towards the bluish direction on the yellow-blue axis of cotton fabric in our research as shown in Fig. 6. In terms of color brightness (C^*^) values, the yellow sample appears brighter with a value of 43.66, while the sample 75B-25Y is less bright with a value of 12.69 compared to other samples. h° represents the hue angle (0° (red)–60° (yellow)–120° (green)–180° (cyan)–240° (blue)–300° (magenta)–360° (red)), primarily used to differentiate between different colors. The h° values of the mixed samples ranged from 110.23 to 220.45. Table 3 displays the color strength data for cotton fabrics that were dyed separately and in various combinations of dyes at different ratios (80:20, 75:25, 50:50, 20:80, and 25:75) in a particular order. The *K/S* values for the blended samples varied from 8.73 to 7.71, with the 80B:20Y sample showing higher *K/S* values compared to the rest of the samples.

**Table 3:** Color study of dyed cotton samples.

Dyes- combination	L^*^	a^*^	b	CV % of K/S	Stdv	Standard Error	K/S	C^*^	H
Blue Dyed sample	24.22	9.40	-28.99	0.86	0.08	0.04	9.38	31.60	286.71
Yellow Dyed sample	89.5	-7.87	70.71	0.37	0.02	0.014	6.65	67.68	84.99
M1 (Blue dye: Yellow dye 80:20)	45.11	-15.29	-8.42	0.80	0.07	0.040	8.73	15.73	220.45
M2 (Blue dye: Yellow dye 75:25)	49.74	-18.59	-1.43	0.24	0.02	0.012	8.62	12.69	187.71
M3 (Blue dye: Yellow dye 50:50)	50.66	-12.32	-11.83	0.61	0.05	0.029	8.43	16.38	155.45
M4 (Blue dye: Yellow dye 25:75)	55.47	-13.83	-8.52	0.79	0.06	0.037	8.11	14.89	120.12
M5 (Blue dye: Yellow dye 20:80)	60.29	-5.96	-15.75	0.27	0.02	0.012	7.71	15.15	110.23

### Leveling and fixation properties of dyed cotton samples

The color variation of the dyed cotton fabric was evaluated by measuring the leveling characteristics at five different locations on the fabric. Measurements were taken at different concentrations ranging from 1 to 4% (o.w.f) at each of these points. It can be observed from Table 4 that the △E values for dyed cotton fabrics with different dye amounts were all less than 1. This result indicates that cotton fabrics showed good leveling properties during scCO_2_ dyeing. It was observed from Table 5 that using different mixtures for the two dyes under study, high fixations ranging from 84.9 to 93.9 were obtained on cotton fabrics. During the dyeing process in scCO_2_, the dye molecules are theoretically attached to the substrate fibers by engaging in a nucleophilic substitution reaction with the functional groups on the macro chains of the substrate fabrics.

**Table 4:** Leveling characteristics of dyed cotton samples.

Mix ratio	1%	2%	3%	4%
∆E _80:20_	0.88	0.71	0.47	0.54
∆E_75:25_	0.75	0.72	0.46	0.51
∆E_50:50_	0.67	0.61	0.44	0.49
∆E_25:75_	0.77	0.65	0.48	0.57
∆E_20:80_	0.81	0.78	0.58	0.60

**Table 5:** Fixation percentage of dyed cotton samples.

Mixed samples	K/S after dyeing	K/S after extraction	Fixation (%)
M1 (Blue dye: Yellow dye 80:20)	8.73	7.55	86.5
M2 (Blue dye: Yellow dye 75:25)	8.62	8.01	92.9
M3 (Blue dye: Yellow dye 50:50)	8.43	7.92	93.9
M4 (Blue dye: Yellow dye 25:75)	8.11	7.32	90.25
M5 (Blue dye: Yellow dye 20:80)	7.71	6.55	84.9

### Colorfastness of dyed cotton samples

All experiments were conducted under optimum conditions using the dyes and their mixtures under study. The optimum scCO_2_ dyeing of fabrics was achieved at (temperature: 120 °C, dye concentration: 3% o.m.f., dyeing time: 90 min, and pressure: 25 MPa). The developed dyes showed outstanding washing durability for staining and fading when using multifiber neighboring fabrics made of cotton, nylon 6, polyester, and wool, graded at 5. This outcome is demonstrated in Table 6, meeting the industry’s international grayscale standards. Our findings were owed to the effective diffusion and penetration of the dyes understudy.

Furthermore, the dyed cotton samples showed outstanding resistance to rubbing (wet and dry) and sweat, achieving a rating of 5. However, the light-fastness exhibited very good outcomes with grades of 4–5. Laundering durability findings showed satisfactory findings following five washing cycles. The results were produced when the combination of dyes interacted significantly with the cotton fibers, potentially creating new chemical bonds between the dyes and cotton. Vinyl sulfone, vinyl chloride, and cyanuric chloride are reactive groups widely utilized in the dyeing of cotton textiles due to their exceptional affinity and permanence. These reactive groups form covalent bonds with the hydroxyl groups in cotton fabrics. Yellow dye undergoes nucleophilic addition reactions with the cellulose hydroxyls, forming stable ether linkages. However, blue dye undergoes nucleophilic substitution reactions with the cellulose hydroxyls. These covalent attachments ensure excellent color fastness (wash, rubbing, perspiration).

**Table 6:** Fastness characteristics of dyed cotton specimen in scCO_2_.

Dye Mixture B: Y	Wash Fastness		Crocking Fastness		Perspiration Fastness				Light Fastness
	Shade	Stain	Wet	Dry	Acidic		Alkali		
					Shade	Stain	Shade	Stain	
Blue Dye	5 (4)	5	5	5	5	5	5	5	4–5
Yellow Dye	5 (4)	5	5	5	5	5	5	5	4–5
M1 (80:20)	5 (4)	5	4–5	5	5	5	5	5	4–5
M2 (75:25)	5 (4)	5	5	5	5	5	5	5	4–5
M3 (50:50)	5 (4)	5	5	5	5	5	5	5	4–5
M4 (25:75)	5 (4)	5	5	5	5	5	5	5	4–5
M5 (20:80)	5 (4)	5	5	5	5	5	5	5	4–5

Note that the fastness units mentioned are represented by integers ranging from 0 to 5. A rating of 0 indicates the lowest fastness, while a rating of 5 indicates the highest fastness. (B, Blue) (Y, Yellow). Furthermore, the test for washing durability involved five continuous cycles of washing.

### Antibacterial activity of both synthesized dyes and dyed samples

**Fig. 7:**
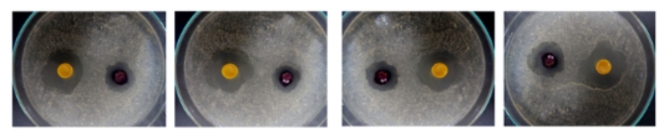
Diameters (mm) of the bacterium colony-clear zones surrounding both dyes.

Table 7 displays the antibacterial effectiveness of the newly produced dyes against four bacterial strains. These include *Staphylococcus aureus* and *Bacillus cereus* as Gram-positive bacteria and *Escherichia coli* and *Pseudomonas aeruginosa* as Gram-negative bacteria. Tetracycline and ciprofloxacin served as comparison drugs. The findings demonstrate that the two dyes show remarkable antibacterial properties, as evidenced by a clear zone surrounding bacterial colonies as shown in Fig. 7. This result could be attributed to the fact that the synthesized dyes may interact better with bacterial or fungal cells than their colloidal form. Subsequently, the molecules can enter the cell membrane, leading to membrane destruction and release of cellular components, ultimately resulting in bacterial death^40,41^.

Table 8 illustrates that all dyed fabrics inhibited the growth of the common bacteria. The dyed samples exhibited a higher antibacterial effect against four strains. The comparison between the antibacterial properties of the dyed cotton samples and the dyes revealed consistent results.

**Table 7:** Antibacterial activity of synthesized dyes.

Dyes	The diameter of the clear zone (mm)			
	Staphylococcus aureus (G+)	Bacillus cereus (G+)	Escherichia coli (G−)	Pseudomonas aeruginosa (G−)
Ciprofloxacin	**24**	**15**	**23**	**17**
Tetracycline	**21**	**14**	**20**	**15**
Blue Dye	18	15	14	13
Yellow Dye	27	24	26	25

**Table 8:** Reduction of bacterial growth of dyed samples.

Substrate	(% Reduction of bacterial growth)			
	Staphylococcus aureus (G+)	Bacillus cereus (G+)	Escherichia coli (G−)	Pseudomous (G−)
Blank sample	0	0	0	0
Blue dyed sample	33.65715	52.71978	6.63897	10.35714
Yellow dyed sample	39.86714	61.4564	7.89469	11.70307
Mixed dyed sample	40.56982	65.96104	7.8564	11.5532

## Conclusion

Previously, the dyeing process of cotton in scCO_2_ was only studied for basic colors, but there is a lack of research on using different color combinations. A combination of different dyes is required to achieve different colors, which is essential for advancing scCO_2_ dyeing, which is integral to the advancement of scCO_2_ dyeing in industries, as the overall look of fabrics is influenced by shades of red, yellow, and blue. In this work, two special reactive disperse dyes were designed; one of them is blue, derived from the anthraquinone parent body, and the other is yellow, incorporating a pyrazole moiety. The dyeing behaviors and ability to mix the colors of two developed reactive disperse dyes on the cotton fabric were examined using dyeing rate curves. The outcomes indicated that there were successful color mixture and other coloration performances on cotton in the scCO_2_ dye bath.

Additionally, system parameters like dye concentration, temperature, pressure, and dyeing duration have notable impacts on absorption behaviors and also ensure proper color coordination at varying concentrations. The dyes under study and their mixtures showed superior performance in terms of washing fastness, with a fading and staining grade of (4–5) and rubbing fastness, with a fading and staining grade of 5. Additionally, they demonstrated very good results in terms of light fastness. Different mixtures of dyes were successfully used for dyeing, showing excellent leveling properties and high fixation. In conclusion, this research provides important information to promote more environmentally friendly manufacturing processes and techniques for supercritical fluid dyeing.

## Electronic supplementary material

Below is the link to the electronic supplementary material.

Supplementary Material 1

## Data Availability

Data is provided within the manuscript or supplementary information files.

## References

[1] AbouElmaaty, T., Abouelenin, S., Elsisi, H. & Okubayashi, S. Eco-friendly Approach for Dyeing Synthetic fabrics with natural dyes using Electron Beam Irradiation. *Fiber Polym.* **23** (3), 759–767 (2022).

[2] Goñi, M. L., Gañán, N. A. & Martini, R. E. Supercritical CO_2_-assisted dyeing and functionalization of polymeric materials: a review of recent advances (2015–2020). (2021). J. CO_2_ Util. 54(July).

[3] Zhou, T., Wang, Y., Zheng, H., Du, B. & Zheng, L. Sustainable and eco-friendly strategies for polyester-cotton blends dyeing in supercritical CO_2_. *J. CO*_*2*_*Util*. **55**, 101816 (2022).

[4] Yan, K., Zhang, Y. Q., Xiao, H., Shi, M. W. & Long, J. J. Development of a special SCFX-AnB3L dye and its application in ecological dyeing of silk with supercritical carbon dioxide. *J. CO*_*2*_*Util.* **35**, 67–78 (2020).

[5] Bai, T., Kobayashi, K., Tamura, K., Jun, Y. & Zheng, L. Supercritical CO_2_ dyeing for nylon, acrylic, polyester, and casein buttons and their optimum dyeing conditions by design of experiments. *J. CO*_*2*_*Util.* **33**, 253–261 (2019).

[6] Abate, M. T. et al. Single-step disperse dyeing and antimicrobial functionalization of polyester fabric with chitosan and derivative in supercritical carbon dioxide. *J. Supercrit Fluids* 147, 231–240 (2019).

[7] Penthala, R. et al. Synthesis of azo and anthraquinone dyes and dyeing of nylon-6,6 in supercritical carbon dioxide. *J. CO*_*2*_*Util.* **38**, 49–58 (2020).

[8] Abou Elmaaty, T., El-Taweel, F. M. & Elsisi, H. G. Water-free dyeing of Polyester and Nylon 6 fabrics with Novel 2-Oxoacetohydrazonoyl cyanide derivatives under a supercritical Carbon Dioxide Medium. *Fiber Polym.* **19** (4), 887–893 (2018).

[9] Abou Elmaaty, T. A., El-Taweel, F., Elsisi, H. & Okubayashi, S. Water-free dyeing of polypropylene fabric under supercritical carbon dioxide and comparison with its aqueous analogue. *J. Supercrit Fluids*. **139**, 114–121 (2018).

[10] Abou Elmaaty, T. A., Sofan, M., Kosbar, T., Elsisi, H. & Negm, I. Green Approach to Dye PET and Nylon 6 fabrics with Novel Pyrazole disperse dyes under supercritical Carbon Dioxide and its aqueous analogue. *Fiber Polym.* **20** (12), 2510–2521 (2019).

[11] Abou Elmaaty, T. et al. Optimization of an eco-friendly dyeing process in both laboratory scale and pilot scale supercritical carbon dioxide unit for polypropylene fabrics with special new disperse dyes. *J. CO*_*2*_*Util.* **33**, 365–371 (2019).

[12] Abou Elmaaty, T., Elsisi, H. & Negm, I. Dyeing characteristics of Polypropylene Fabric Dyed with Special Disperse dyes using supercritical Carbon Dioxide. *Fiber Polym.* **22** (5), 1314–1319 (2021).

[13] Cheng, Y. W. et al. Synthesis of Azo disperse dyes with high absorption for efficient polyethylene terephthalate dyeing performances in Supercritical Carbon Dioxide. *Polym* 14(15) (2022). 10.3390/polym14153020 PMC9331285 35893983

[14] Penthala, R. et al. An ecofriendly dyeing of nylon and cotton fabrics in supercritical CO_2_ with novel tricyanopyrrolidone reactive disperse dye. J. CO_2_ Util. 60, 102004 (2022). (2022).

[15] Abou Elmaaty, T. et al. Pilot scale water free dyeing of pure cotton under supercritical carbon dioxide. *Carbohydr. Polym. Techno Appl.* 1,100010 (2020).

[16] Abou, T., Elsisi, H., Negm, E., Ayad, S. & Sofan, M. Novel nano silica assisted synthesis of azo pyrazole for the sustainable dyeing and antimicrobial finishing of cotton fabrics in supercritical carbon dioxide. *J. Supercrit Fluids*. **179**, 105354 (2021).

[17] Abou Elmaaty, T., Sofan, M., Ayad, S., Negm, E. & Elsisi, H. Novel synthesis of reactive disperse dyes for dyeing and antibacterial finishing of cotton fabric under scCO_2_. *J. CO*_*2*_*Util.* 61(May),102053 (2022).

[18] Gao, D., Cui, H. S., Huang, T. T., Yang, D. F. & Lin, J. X. Synthesis of reactive disperse dyes containing halogenated acetamide group for dyeing cotton fabric in supercritical carbon dioxide. *J. Supercrit Fluids*, 86, 108–114 (2014).

[19] Luo, X. et al. Novel sustainable synthesis of dyes for clean dyeing of wool and cotton fibres in supercritical carbon dioxide. *J. Clean. Prod.* 199, 1–10 (2018).

[20] Yang, D. et al. ting,xin. Dyeing of cotton fabric with reactive disperse dye contain acyl fluoride group in supercritical carbon dioxide. Dyes Pigments 139, 566–574 (2017). (2017).

[21] Gong, D., Jing, X., Zhao, Y., Zheng, H. & Zheng, L. One-step supercritical CO_2_ color matching of polyester with dye mixtures. *J. CO*_*2*_*Util.* **44**, 101396 (2021).

[22] Huang, G., Dai, J., Dong, F., Wang, J. & Jia, Y. Compatibility of a disperse dye mixture in supercritical carbon dioxide dyeing. *Color. Technol.* **129** (4), 305–311 (2013).

[23] Wang, Y., Jing, X., Zhao, Y., Zheng, L. & Zheng, H. Waterless beam dyeing in supercritical CO_2_: establishment of a clean and efficient color matching system. *J. CO*_*2*_*Util.* **43**, 101368 (2021).

[24] Zhao, X. Y. et al. Investigation of the uptake and compatibility behaviors of special disperse dyes developed for sustainable color matching in supercritical carbon dioxide. *J. CO*_*2*_*Util.* 72, 102478 (2023).

[25] Fan, Y., Zhang, Y. Q., Yan, K. & Long, J. J. Synthesis of a Novel disperse reactive dye involving a versatile Bridge Group for the sustainable coloration of natural fibers in supercritical Carbon Dioxide. *Adv. Sci.* **6** (1), 1801368 (2019). 10.1002/advs.201801368 PMC6325576 30643724

[26] Salabert, J., Sebastián, R. M. & Vallribera, A. Anthraquinone dyes for superhydrophobic cotton. *Chem. Commun.* **51** (75), 14251–14254 (2015). 10.1039/c5cc06028a 26265296

[27] Shan, B. et al. A new kind of H-acid monoazo-anthraquinone reactive dyes with surprising colour. *Dyes Pigm.*, 123, 44–54 (2015).

[28] Zielske, A. G. (Tosyloxy)anthraquinones: Versatile Synthons for the Preparation of Various Aminoanthraquinones. J. Org. Chem. 52(7), 1305–1309 (1987). (1987).

[29] Abou Elmaaty, T., Elnagar, K., Hassan, S. & Gamal, H. Antibacterial activity and dyeing characteristics of some azo-pyazole disperse dyes using eco-friendly ultrasound energy for PET fabric. *Int. J. Sci. Eng. Res.* **5** (5), 1156–1161 (2014).

[30] Karci, F., Şener, N., Yamaç, M., Şener, I. & Demirçali, A. The synthesis, antimicrobial activity and absorption characteristics of some novel heterocyclic disazo dyes. *Dyes Pigm.* 80(1), 47–52 (2009).

[31] Kotla, V. V., Dalavai, V. K. & Chunduri, V. R. Synthesis and biological activity studies of some novel pyrazoline derivatives. *Der Pharma Chem.* **4** (5), 2003–2008 (2012).

[32] Metwally, M. A., Bondock, S., El-Desouky, S. I. & Abdou, M. M. A Worthy Insight into the dyeing applications of Azo Pyrazolyl Dyes. *Int. J. Mod. Org. Chem.* 1(3), 165–192 (2012).

[33] Sayed, A. Z., Aboul-Fetouh, M. S. & Nassar, H. S. Synthesis, biological activity and dyeing performance of some novel azo disperse dyes incorporating pyrazolo[1,5-a]pyrimidines for dyeing of polyester fabrics. *J. Mol. Struct.* **1010**, 146–151 (2012).

[34] Kan, C. W. & Fong, K. W. F. A study of reusing vinyl sulfone based reactive dye for dyeing cotton fiber. *Fiber Polym.* 18(11), 2176–2186 (2017).

[35] Kumar Samanta, A. Advanced Methods and Tools for Color Measuring and Matching: For Quality Check of Colored Products of Textiles and Apparel Industry. In Advances in Colorimetry [Working Title].

[36] Amutha, K. A practical guide to Textile Testing. In A practical guide to Textile Testing (1st Editio). WPI Publishing.

[37] AATCC - Technical Manual. In American Association of Textile Chemists and Colorists (Vol. 80). (2005).

[38] Sofan, M., Abou Elmaaty, T., Hashem, N. & Zheng, L. One-step dyeing and Antimicrobial Finishing of PET Fabric with Novel 4-Arylazopyrazolone disperse dyes. *Fiber Polym.* 21, 2817–2826 (2020).

[39] Long, J. J. et al. Dyeing of cotton fabric with a reactive disperse dye in supercritical carbon dioxide. *J. Supercrit Fluids* 69,13–20 (2012).

[40] Qun, T. et al. Antibacterial activities of anthraquinones: structure-activity relationships and action mechanisms. *RSC Med. Chem.* 14, 1446–1471 (2023). 10.1039/d3md00116d PMC10429894 37593578

[41] Rizk, H. F., Ibrahim, S. A. & El-Borai, M. A. Synthesis, fastness properties, color assessment and antimicrobial activity of some azo reactive dyes having pyrazole moiety. Dyes Pigments 112, 86–92(2015). (2015).

