# Supplementary material for: A green approach for dyeing cotton fabrics using synthesized reactive disperse dyes and their mixtures under supercritical CO2 medium
Source: Sci Rep. 2024 Nov 6;14:26887. doi: 10.1038/s41598-024-77606-0 (PMC11541500; doi:10.1038/s41598-024-77606-0)

**A Green Approach for Dyeing Cotton Fabrics Using Special Dyes and Their Mixtures Under a Supercritical CO_2_ Dyeing Medium**

**Hanan Elsisi^1*^, Shahinaz Abouelenin^1^, Tarek Abou Elmaaty^1^,** **Elham Negm^2^**

^1^Department of Textile Printing, Dyeing & Finishing, Faculty of Applied Arts, Damietta University, Damietta 34512, Egypt

^2^Chemistry Department, Faculty of Science, Damietta University, New Damietta 34517, Egypt

**^1^H-NMR data:**

**Compound 3 (Blue Dye)**


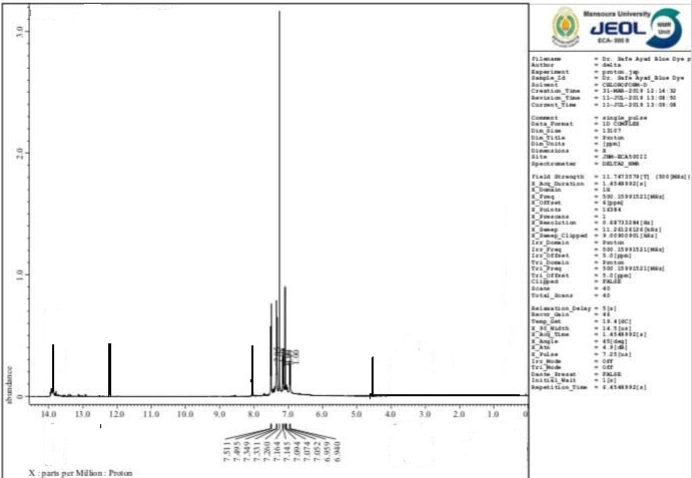


**^13^C-NMR data:**
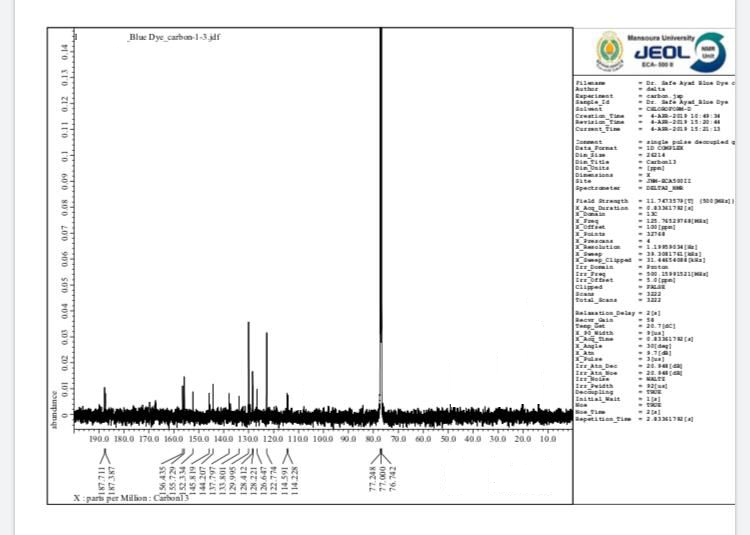


**Mass spectra**


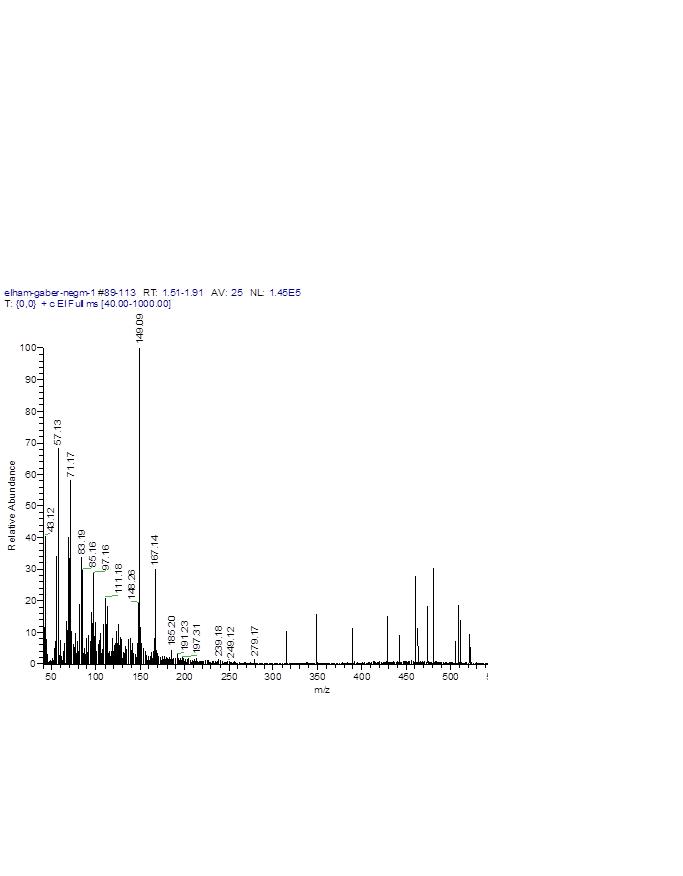


**^1^H-NMR data:**

**Compound 5**


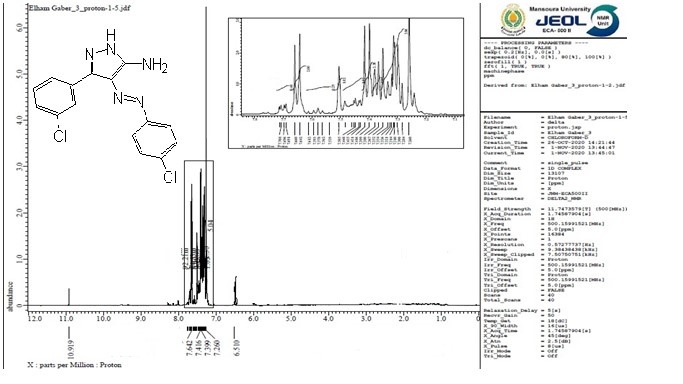


**^1^H-NMR (D_2_O) data:**


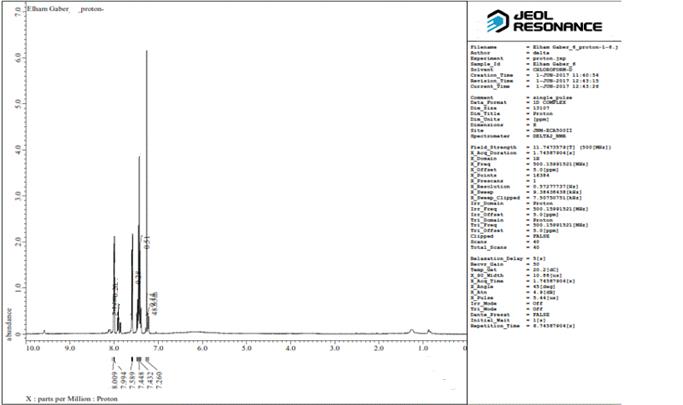


**^13^C-NMR data:**


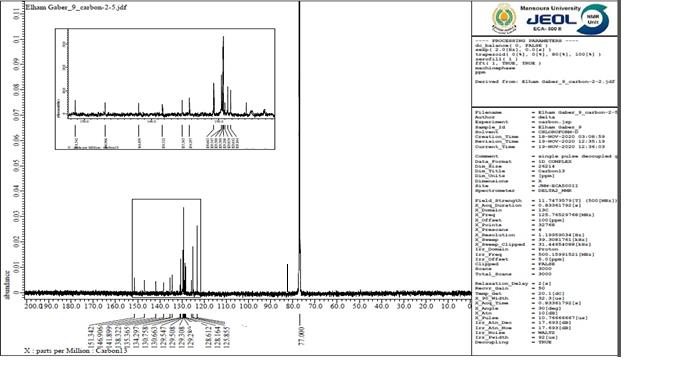


**Mass spectra**


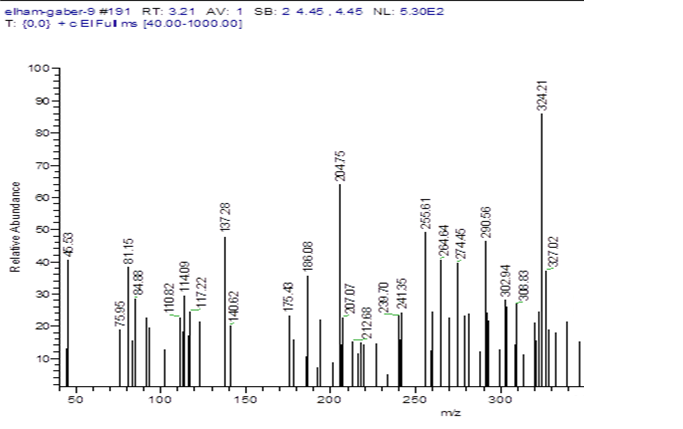


**^1^H-NMR data:**

**Compound 6 (Yellow Dye)**


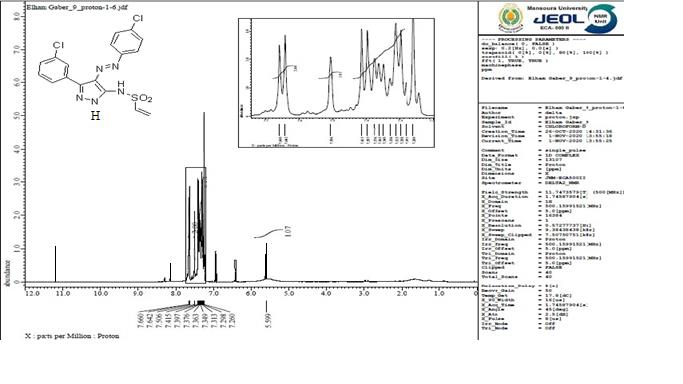


**^1^H-NMR (D_2_O) data:**


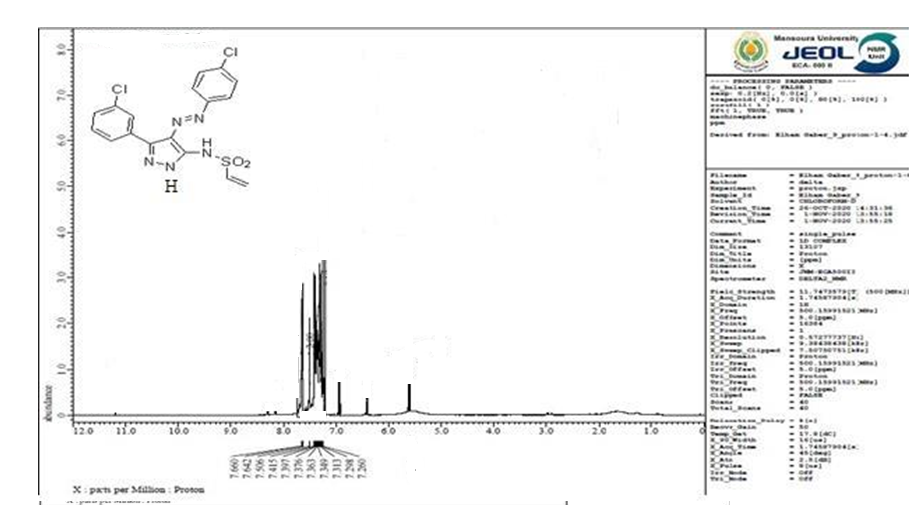


**^13^C-NMR data:**


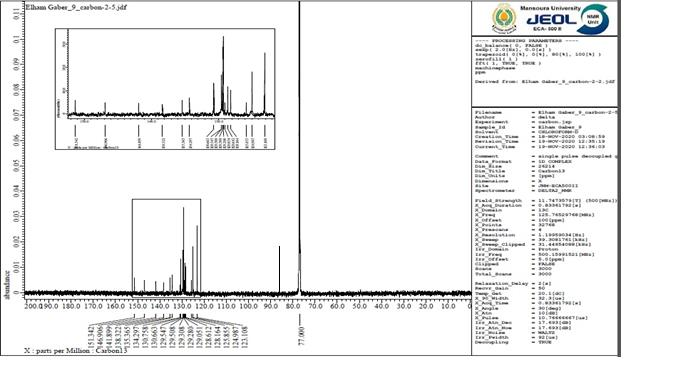


**Mass spectra**


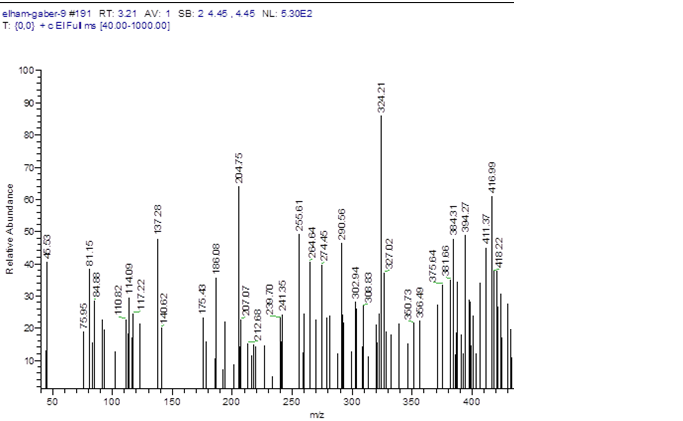

Supplement: Supplementary file 1 — Supplementary Material 1 [file 41598_2024_77606_MOESM1_ESM.docx]
